# Supplementary material for: Grassland intensification effects cascade to alter multifunctionality of wetlands within metaecosystems
Source: Nat Commun. 2023 Dec 13;14:8267. doi: 10.1038/s41467-023-44104-2 (PMC10719369; doi:10.1038/s41467-023-44104-2)
Supplement: Supplementary file 1 — Supplementary Information [file 41467_2023_44104_MOESM1_ESM.pdf]

Supplementary Information (SI)

Table of Contents

- **Supplementary Note 1**
- **Supplementary Note 2**
- **Supplementary Note 3**
- **Supplementary Tables**
- **Supplementary Figures**
- **Supplementary References**

Supplementary Note 1

Study site description

Our study area is the Archbold Biological Research Station's Buck Island Ranch (BIR), located in south-central Florida (27°09'N, 81°11W). BIR is a commercial cow-calf production ranch and a long-term agro-ecological field station. The total area of BIR is 4,252 ha, which was dominated by wet prairie (1,771 ha), dry prairie (1,604 ha), and wetland (544 ha) [1]. The landscape of Florida prairie ecosystems has adapted to natural fire regimes at 1- to 4-years return intervals. Thus, most pasture areas in BIR are prescribed burned every 2 to 3 years to maintain the pyrogenic system as well as to manage forage. BIR has a humid subtropical climate with distinct hot wet (June – October) and cool dry (November – May) seasons. Average daily temperatures during the two seasons are 26.1°C and 19.4 °C, respectively. Average annual precipitation is ~1,300 mm, with approximately 70% falling during the wet season.

After being heavily drained and converted to forage grass (*Paspalum notatum* Flugge) in the 1960s, the central and north-central areas of BIR are classified as Intensively managed (IM) pastures (**Fig. S1**). IM pastures also received nitrogen (N), phosphorus (P), and potassium (K) fertilizers (although P and K fertilization were up until 1986), regular lime, and wet-season intense grazing activities during the wet season starting in the 1970s. N fertilization is still being applied using NH_4SO_4 or NH_4NO_3 at a rate of 56 kg/ha annually or semiannually, while P and K were historically applied using P_2O_5 and K_2O at a rate of 34 – 90 kg/ha [2]. Cattle density in IM pastures is 347 animal days ha^{-1} yr^{-1}. IM pastures are located at elevation of ~7.9 to 10.7 m above sea level, gently sloping from north to the south and draining into Harney Pond Canal. Soils in IM pastures are mainly spodosols and alfisols [2]

The prairies and savannas surrounding IM pastures in BIR are less drained, never fertilized, only partially converted to *P. notatum*, and moderately grazed during the dry seasons, which are classified as Semi-natural (SN) (i.e., less intensively managed) pastures (**Fig. S1**). In addition to *P. notatum*, other common plant species in SN pastures are bunchgrasses (*Andropogon virginicus*, *Axonopus affinis*, and *Panicum longifolium*), forbs (*Lachnanthes caroliniana* and *Eupatorium mohrii*), and woody dicots (*Eupatorium capillifolium* and *Euthamia graminifolia*). SN pastures are located at elevation of ~8.0 to 10.0 m above sea level. Within SN pastures, in the area north of the Harney Pond Canal, drainage is gradually to the southeast, while in the area south of the Harney Pond Canal, the water drains from south to north. Cattle density in SN pastures is 152 animal days ha^{-1} yr^{-1}. Soils in SN pastures are primarily alfisols and spodosols. These two IM and SN pasture types represent the two dominant and most widely adopted ranch managing regimes in south Florida and southeastern U.S. [3], and are also typical of many managed grasslands in other subtropical and tropical biomes worldwide.

Both types of pastures include many small wetlands that are seasonally flooded, emergent or shrub freshwater marshes [4]. These wetlands were historically considered as isolated because they were geographically distinct and separated from other aquatic bodies. However, with the construction of extensive ditches, some wetlands are connected via ditches during the wet seasons, especially in IM pastures. BIR has a total of more than 600 such wetlands with a mean size of 0.87 ha and hydroperiod from 2 to 10 months. These wetlands are scattered across the

ranch and represent ~15% of total land area, which is typical for the Lake Okeechobee Basin [5]. Cattle have free access to the wetlands within the same pasture for cooling and feeding. Embedded wetlands are exposed to prescribed burns ignited in pastures and sometimes get burned when conditions are sufficiently dry. Owing to influences of upland management practices, wetlands located in different pasture types are shown with distinct biotic and abiotic attributes and thereby classified as IM and SN wetlands. For example, dominant plants in IM wetlands include *Juncus effusus*, *Pontedaria cordata*, and *Panicum hemitomon*. In SN wetlands, common plant species are more diverse, which include *Pontedaria cordata*, *Panicum hemitomon*, *Amphicarpum muehlenbergianum*, *Justicia angusta*, *Sagittaria lancifolia*, and *Bacopa caroliana*.

Supplementary Note 2

Existing experimental infrastructure

Since 2001, the BIR has been an active location for research on ecosystem services provided by subtropical grasslands and wetlands. There is a dense sensor network installed at the BIR, including five eddy covariance flux towers, 32 groundwater wells, and many water sampling locations. BIR has accumulated co-located long-term datasets (e.g., climate, plant community and functional traits, productivity, soil nutrients, hydrology, GHG fluxes, phenology). These datasets serve as the foundational baseline to understand the long-term agro-ecological responses and dynamics of subtropical grasslands and wetlands to different management regimes.

In addition to the long-term monitoring, BIR has several existing experimental infrastructures available for research. These experimental infrastructures also demonstrate the Archbold BIR's commitment and interests in long-term agricultural and ecological research, as well as contributions to cross-site comparisons and large-scale syntheses. The first one is the sixteen 16-ha experimental pastures established in 2016 as part of the U.S. Department of Agriculture (USDA) NIFA funded project (2016-2020). These experimental grassland units were originally designed to assess the interaction of management intensity and grazing-fire pattern on greenhouse gas flux, water use efficiency, forage productivity and quality and plant diversity. The experiment was set up as a randomized block design with two blocks crossing two pasture types (Intensively managed and Semi-natural). The second infrastructure is the 40 wetlands with fire and grazing treatments imposed on the two types of wetlands. This experiment, established in 2006 and was also originally funded by the USDA from 2006-2009, aimed to understand the interactive effects of upland management intensification, grazing and fire disturbances on wetland communities and ecosystems. This wetland experiment is a randomized block design with a full factorial of 2×2×2 crossing pasture management (Intensively managed and Semi-natural wetlands), grazing (grazed and not grazed), and prescribed fire (burned and unburned). There are 5 replicates of each treatment (i.e., 8 unique treatment combination). The current study took advantage of this experimental design and leveraged previously collected data by only analyzing the wetland embedded in Intensively managed and Semi-natural grasslands within a subset of grazed wetlands to estimate the effects of upland intensification.

Supplementary Note 3

Grassland datasets description

Grassland soil nutrient data: Soils were collected in eight NIFA pastures in November 2016 and November 2019. Four pastures were Intensively managed pastures and the other four were Semi-natural pastures. We collected nine soil samples per pasture (i.e., 3 per pasture-third) from random points that were at different locations for each of the two years. Soil cores that were analyzed for nitrogen (N), phosphorus (P), and organic matter were taken at 0-15 cm deep with a push core of 3 cm in diameter. Upon return from the field, soil samples were oven dried and passed through a 2 mm sieve. Soil organic matter was measured by loss-on-ignition by ashing 5 g of oven dried samples under 450 °C for 16 hours. Total P and Mehlich-3 P concentrations were determined by Aqua Regia Extraction and Double Acid Extraction followed by spectrophotometer reading (Biotek Epoch 2), and soil inorganic N (nitrate and ammonium) concentrations were determined with 2M KCl extraction and analyzed with a SEAL analytical segmented flow autoanalyzer (WI, USA). In addition, during soil sieving, roots (i.e., rhizomes, coarse roots, and fine roots) were separated and collected to determine root biomass. Soil cores that were used to determine total carbon (C) and nitrogen (N) were from 0-10 cm deep. These cores were collected using a hammer core that was ~7.5 cm in diameter. Soil samples were analyzed for bulk density and total C and N, which were determined using a LECO CN628 C/N Determinator (LECO Corporation, MI).

Grassland ditch water quality data: Internal ditches within grasslands were monitored for total P, ortho-phosphate (PO_4^{3-}), NH_4^+, NO_3^-, and total N monthly from 2003 – 2015. Four ditches were in Intensively managed pastures and the other four were in Semi-natural pastures. Water samples were collected with a 1 Liter dipping pole. The bottle is immersed upside down in water and rinsed 3 times before collecting a sample. Equipment blanks and field duplicates were collected for each sampling event. Samples for ortho-phosphate were filtered after sampling and were analyzed within 48 hours of sample collection using malachite green. Ammonia and nitrate were analyzed within 21 days of sampling. Grab samples analyzed for total P and total N were preserved with 50% H_2SO_4. A spectrophotometer (Biotek Epoch 2) was used to analyze these samples.

Grassland plant biodiversity data: The vegetation of each of the eight NIFA pastures, four Intensively managed and four Semi-natural, was surveyed annually between 2016 and 2019. Surveys took place in September-October each year. Vegetation surveys were accomplished by selecting 15 randomly located 1-m^2 circular plots in each of the three patches (third of a pasture) within each of the eight pastures, totaling 45 plots per pasture. We recorded all vascular plant species present in each plot. In 2018 and 2019, we also recorded species canopy cover (%) to the nearest percent.

Using this dataset, we calculated plant species richness (total, native and non-native) in each patch (i.e., by combining data from the 15 plots), in each pasture (i.e., by combining data from the 45 plots), for each sampling event. We also calculated the exponential of Shannon diversity and plant alpha diversity (H', Jost 2007) in each patch using the incidence of each species in each patch (i.e., how many times a given species occurred in the 15 plots), and in each pasture

(i.e., how many times a given species occurred in the 45 plots). Plant beta diversity was calculated based on the approach from [6].

Grassland game camera biodiversity data: For the period of 2016-01-01 to 2018-12-31, a game camera array of 44 cameras (17 in Intensively managed and 27 in Semi-natural pastures) were evenly distributed (1 km grid) across the Buck Island Ranch. Each camera was run continuously and when triggered collected a burst of 10 images, 1 per second, after which a 5-minute forced quite period was invoked before camera could be triggered again. Identification of wildlife was assisted by machine learning [7], and multiple human observers for images unidentified by automated processes. The initial collection of this dataset was focused on questions surrounding feral invasive pigs, as such certain groups of vertebrates are only identified to higher levels, for example most bird were pooled as bird with exception of quail and turkey.

For the analyses, the image dataset was summarized based on a count of number of days each species was recorded per month for each of the 44 cameras. The wildlife dataset included 58,122 observations of species occurring per day. Summaries of species or taxon levels used the average monthly occurrence of each species encountered for the 3 years for each camera.

Grassland primary productivity data: We measured aboveground net primary productivity (ANPP, i.e., the quantity of biomass produced on an annual basis) using the moveable exclosure (ME) method [8,9] with small exclosures (> 0.25 m^2) randomly located within each pasture to prevent cattle grazing and paired with same-sized grazed plots (paired plots (PP)) located next to the exclosures. Within a pasture, we setup three exclosures in one of the pasture-thirds, because we assumed each third of a pasture would be similar, because they were all under the same fire regime. Six NIFA pastures were selected for ANPP measurements, 3 Intensively managed and 3 Semi-natural. Sampling occurred in late 2016 before the start of the experiment and when cattle were rotated out of a pasture (~3-4 times a year). Sampling consisted of clipping and harvesting the biomass that was produced within the exclosure and directly outside the exclosure (paired grazed plots) in separate paper bags. If woody species were found within plots, only new growth was harvested. Biomass was then dried to constant mass at 50-60°C usually for about 2-3 days in a drying oven (Thermo Scientific, Heratherm OMH750) and dry weight was measured using a precision balance (Denver instrument, XL-6100).

Based on these measurements, we calculated ANPP [8], following ANPP = (Σ ME(T_2) – PP (T_1)) + residual biomass in ME at end of growing season, where ME = exclosure and PP = grazed paired plot. T_1 = time 1 and T_2= time 2. ANPP is an indicator of forage production and an indicator of the amount of forage available for cattle. Detailed sampling and analysis can be found in Boughton et al., (2022).

Grassland forage quality data: In 2017, we collected forage in 3 randomly located 0.25 m^2 circular plots within one pasture-third (N= 3 per pasture) every other month. Biomass was clipped to ground level and did not include litter. If woody species were found within plots, only new growth was harvested. Detailed sampling and analysis can be found in Boughton et al., (2022). Specifically, after drying and weighing, we ground the biomass using a Wiley mill to fit through a 1-mm screen (Thomas Scientific, Model 4). These samples were sent to the University

of Florida Forage Evaluation Support Laboratory (IFAS – FESL) for forage quality analysis. We obtained total P, total N (%) and *in vitro* Organic Matter Digestibility (IVDMD, %) for each sample. The method used for total P and total N is a modification of the standard Kjeldahl procedure. Samples were digested using a modification of the aluminum block digestion procedure of [11]. Sample weight was 0.25 g, catalyst used was 1.5 g of 9:1 K_2SO_4:$CuSO_4$, and digestion was conducted for at least 4h at 375°C using 6 ml of H_2SO_4 and 2 ml H_2O_2. P or N in the digestate was determined by semi-automated colorimetry [12]. The procedure used to obtain IVDMD is "two-stage" [13]. First, forage samples were incubated with rumen microorganisms for 48 h followed by incubation with acid-pepsin. Results are expressed in the percentage units, meaning that the percentage of organic matter which was "digested" for each measured indicator (https://agronomy.ifas.ufl.edu/department-labs/forage-evaluation-support-laboratory/in-vitro-organic-matter/).

Starting in January 2018, we sampled plant biomass within each pasture once a month (all 8 pastures). We used a small quadrat ($0.1m^2$) to standardize the area sampled in each pasture. We sampled biomass at four random locations within one pasture third and these four samples were composited into one sample. Biomass was placed within paper bags and labelled with pasture and patch information and date. Similar to 2017 samples, we dried the biomass to constant mass and ground it using a Wiley mill (Thomas Model 4 Wiley Mill 1188Y51). Samples were sent to the University of Florida Forage Evaluation Support Laboratory (IFAS – FESL) for analysis. We obtained total P, total N (%), and *in vitro* Organic Matter Digestibility (IVDMD, %) for each sample following the same protocol described above.

Grassland gas fluxes data: The net grassland exchange of CO_2 and CH_4 was determined using the eddy covariance (EC) technique, which were assessed in other two pastures, Griffin Park as an IM pasture and Winter #6 as a SN pasture, where the EC towers were installed in its center. Data used from this study was collected between July 2013 and October 2015 at 30-min intervals. Negative values indicated uptake and positive values indicated emission from ecosystems. Gaps in the half-hour CO_2 flux record were filled using the Eddy covariance gap-filling and flux partitioning online tool (http://www.bgc-jena.mpg.de/~MDIwork/eddyproc/index.php). This tool fills gaps in CO_2 records using the Look-Up table and mean diurnal course methods, and partitions CO_2 fluxes into ecosystem respiration (R_{eco}) and gross primary production (GPP). Half-hour CH_4 gaps of < 2.5 h were filled using a linear interpolation, and gaps of > 2.5 h were filled using the mean diurnal variation method. Missing CH_4 values for a specific half-hour were replaced by the mean of that specific half-hour of four adjacent days. Cumulative monthly values within each pasture were used in this analysis. We also calculated overall cumulative GHG fluxes over the entire sampling period that the SN pasture had –807.72 g CO_2 per m^2 and 58.14 g CH_4 per m^2, whereas the IM pasture had –348.71 g CO_2 per m^2 and 120.87 g CH_4 per m^2. More technical details can be found in Paudel et al., (2023) and Gomez-Casanovas et al., (2018).

Grassland animal use data: Cattle use was monitored at the pasture scale for the entire ranch. Animal use days were recorded for each pasture, by recording the number of animals and the in and out days for each pastures. Stocking density was calculated by dividing the number of animal use days by the pasture size.

Wetland datasets description

Wetland soil nutrient data: Wetland soil samples were collected in 2007, 2009, and 2016 at 0-15 cm depth. The soil nutrient and carbon content have been analyzed in Ho et al. (2018). Briefly, in each wetland ~200 g soil was sampled, which were oven dried at 70 °C for 72 h, ground using mills, then analyzed for the nutrient content and other basic features. Soil total carbon (TC) and total nitrogen (TN) were analyzed on either a Elementar Vario Micro Cube or Carlo Erba Strumentazione NA 1500C/H/N Analyzer. Soil total phosphorus (TP) was analyzed using the EPA method 365.1 Rev. 2 on an AQ2 Discrete Analyzer. Soil organic matter (OM) was analyzed using the loss-on-ignition method by dry ashing at 550 °C for 4 h. In addition, soil cores (0-50 cm) were collected in 2005, and sieved (2-mm) to separate and collect fine and coarse roots for determining root biomass.

Wetland water quality data: Wetland water samples for nutrient analysis were collected at the end of wet seasons in 2006, 2008, 2009, and 2014. Three grab samples were taken using acid washed 100 ml bottle and a sampling pole in each wetland at the wetland center (the central staff gauge), 10 m from the wetland center, and 2-3 m from the wetland edge, respectively. Fresh water samples were preserved with H_2SO_4 in a 4 °C fridge, then tested for chemical characteristics through spectrophotometry. Variable values at three sampling sites were averaged within each wetland and year. More sampling and analysis details were given in Jansen et al. (2019).

Wetland vegetation data: Wetland plant surveys were conducted between October – November in 2018. The species presence/absence and occurrence frequency were collected using 1-m^2 circular quadrats at 15 random points per wetland stratified by five zones (center, and NW, NE, SW, and SE quadrants). Plant community variables were averaged within each wetland. The sampling approach was detailed and consistent with Boughton et al., (2016).

Wetland primary productivity was sampled in 2016 and 2017. Original vegetation was clipped at the beginning of growing seasons during March and April. Then a 0.25 m^2 exclosure used for avoiding cattle grazing was placed in five stratified zones in each wetland. Finally, the newly grown vegetation in the 0.25 m^2 plots was harvested, dried, and weighed at the peak growing season in September. More sampling and analysis details were given in Sonnier et al., (2020).

Wetland vertebrate and invertebrate data: Wetland aquatic ectothermic vertebrates were sampled in September 2006 when seasonal wetlands were flooded for at least 2 weeks, and in the middle of the growing season. Minnow traps were placed in the water 10-12 cm deep in 16 random points along the edge of each wetland. Vertebrate samples were collected and identified the following day and then released.

Wetland invertebrates were sampled in September 2006 in five stratified random points in each wetland. At each sampling point, two 1 m-long sweeps with D-frame nets (~0.05 m^2, 0.5mm mesh) were conducted to collect invertebrates. Invertebrate samples were preserved in 70% isopropanol in fields then transported to the lab for identification. More details could be found in Medley et al., (2015).

Wetland CH_4 fluxes data: Wetland net CH_4 fluxes were sampled in the dry (between November and March) and wet (between July and September) seasons from 2013 to 2015 using a closed chamber technique and an open path CH_4 analyzer (LI-7700, LI-COR, Inc., Lincoln, NE USA) in 16 wetlands. In each wetland, six sampling sites, two from the shallow area, two from the intermediate area and two from the deep area, were randomly selected throughout the wetland regardless of vegetation type and canopy height. Details about the chamber design and measuring process could be found in DeLucia et al., (2019).

Supplementary Tables

Table S1. Main differences between Intensively managed and Semi-natural grasslands, where a portfolio of integrated practices was implemented. Land-use intensification effects refer to the differences in management practices between Semi-natural vs. Intensively managed grasslands

Semi-natural grasslands	Intensively managed grasslands
1. No fertilization	1. N, P, K (P and K were up until 1986) fertilizers received
2. Partially converted to non-native forage grasses	2. Completely converted to non-native forage grasses (e.g., Bahiagrass)
3. No lime application	3. Lime applied
4. Constructed less extensive drainage ditches	4. Constructed extensive drainage ditches
5. No water retention infrastructure	5. Included water retention infrastructure (e.g., riser board)
6. Introduced light cattle grazing in dry seasons	6. Introduced heavy cattle grazing in wet seasons

Table S2. Effects of land-use intensification tested using the one-sided Kruskal-Wallis on grassland and embedded wetland multifunctional indexes as quantified by four different approaches: (1) Simple averaging; (2) Services-based weighted averaging; (3) Euclidean cluster-based weighted averaging, and (4) Top 50% quantile threshold

**Ecosystem** / **Approach**	**Grassland**		**Wetland**	
	**Chi-sq**	***P***	**Chi-sq**	***P***
Simple averaging	2.08	0.15	1.29	0.26
Service-based weighted averaging	3	0.08	5.49	0.02*
Cluster-based weighted averaging	5.33	0.02*	1.65	0.20
50% quantile threshold	5.89	0.02*	5.23	0.02*

Table S3. Model specification of best models for each response variable of ecosystem function and service and overall model performance that includes AIC, BIC, marginal and conditional R^2

<table>
<thead>
<tr><th>Dataset</th><th>Indicator</th><th rowspan="2">AIC</th><th rowspan="2">BIC</th><th rowspan="2">Marginal R^2</th><th rowspan="2">Conditional R^2</th></tr>
<tr><th colspan="2">Model specification</th></tr>
</thead>
<tbody>
<tr><td colspan="6">Grasslands best models</td></tr>
<tr><td>Grassland soil</td><td>TN</td><td>203.81</td><td>218.27</td><td>0.391</td><td>0.391</td></tr>
<tr><td colspan="6">lmer(log(value) ~ Pasture_Type + Elevation + (1|Year), data = pas.soil.tn)</td></tr>
<tr><td>Grassland soil</td><td>NH_4^+</td><td>178.32</td><td>189.76</td><td>0.155</td><td>0.155</td></tr>
<tr><td colspan="6">lm(sqrt(value) ~ Pasture_Type + Elevation, data = pas.soil.nh4)</td></tr>
<tr><td>Grassland soil</td><td>NO_3^-</td><td>536.43</td><td>557.07</td><td>0.055</td><td>0.411</td></tr>
<tr><td colspan="6">lmer(log(value+0.01) ~ Pasture_Type + Elevation + (1+Pasture_Type|Year), data = pas.soil.no3)</td></tr>
<tr><td>Grassland soil</td><td>TP</td><td>173.44</td><td>188.29</td><td>0.357</td><td>0.357</td></tr>
<tr><td colspan="6">lmer(log(value) ~ Pasture_Type + Elevation + (1|Year), data = pas.soil.tp)</td></tr>
<tr><td>Grassland soil</td><td>Mehlich3-P</td><td>227.18</td><td>241.85</td><td>0.039</td><td>0.546</td></tr>
<tr><td colspan="6">lmer(log(value) ~ Pasture_Type + Elevation + (1|Year), data = pas.soil.mp)</td></tr>
<tr><td>Grassland soil</td><td>C/N ratio</td><td>624.32</td><td>638.77</td><td>0.074</td><td>0.145</td></tr>
<tr><td colspan="6">lmer(value ~ Pasture_Type + Elevation + (1|Year), data = pas.soil.cn)</td></tr>
<tr><td>Grassland soil</td><td>OM</td><td>189.03</td><td>203.81</td><td>0.538</td><td>0.538</td></tr>
<tr><td colspan="6">lmer(log(value) ~ Pasture_Type + Elevation + (1|Year), data = pas.soil.om)</td></tr>
<tr><td>Grassland soil</td><td>TC</td><td>206.84</td><td>221.37</td><td>0.341</td><td>0.341</td></tr>
<tr><td colspan="6">lmer(log(value) ~ Pasture_Type + Elevation + (1|Year), data = pas.soil.tc)</td></tr>
<tr><td>Ditch water</td><td>TN</td><td>251.63</td><td>277.57</td><td>0.028</td><td>0.097</td></tr>
<tr><td colspan="6">lmer((value)^(1/3) ~ Pasture_Type + Elevation + (1|Year) + (1|Month), data = pas.water.tn)</td></tr>
<tr><td>Ditch water</td><td>NH_4^+</td><td>2822.0</td><td>2844.9</td><td>0.011</td><td>0.174</td></tr>
<tr><td colspan="6">lmer(log(value+0.001) ~ Pasture_Type + Elevation + (1|Year), data = pas.water.nh4)</td></tr>
<tr><td>Ditch water</td><td>NO_3^-</td><td>582.84</td><td>608.78</td><td>0.006</td><td>0.192</td></tr>
<tr><td colspan="6">lmer((value)^(1/3) ~ Pasture_Type + Elevation + (1|Year) + (1|Month), data = pas.water.no3)</td></tr>
<tr><td>Ditch water</td><td>TP</td><td>-504.67</td><td>-470.48</td><td>0.087</td><td>0.227</td></tr>
<tr><td colspan="6">lmer((value)^(1/3) ~ Pasture_Type + Elevation + (1+Pasture_Type|Year), data = pas.water.tp)</td></tr>
<tr><td>Ditch water</td><td>PO_4^{3-}</td><td>3182.3</td><td>3216.5</td><td>0.038</td><td>0.209</td></tr>
<tr><td colspan="6">lmer(log(value+0.001) ~ Pasture_Type + Elevation + (1+Pasture_Type|Year), data = pas.water.op)</td></tr>
<tr><td>Grassland plant</td><td>Total richness</td><td>287.59</td><td>296.94</td><td>0.497</td><td>0.650</td></tr>
<tr><td colspan="6">lmer(value ~ Pasture_Type + Elevation + (1|Year), data = pas.plant.rich)</td></tr>
<tr><td>Grassland plant</td><td>α-diversity</td><td>12.05</td><td>19.38</td><td>0.789</td><td>0.789</td></tr>
<tr><td colspan="6">lmer(log(value) ~ Pasture_Type + Elevation + (1|Year), data = pas.plant.shan)</td></tr>
<tr><td>Grassland plant</td><td>β-diversity</td><td>-182.35</td><td>-169.25</td><td>0.340</td><td>0.459</td></tr>
<tr><td colspan="6">lmer(sqrt(value) ~ Pasture_Type + Elevation + (1+Pasture_Type|Year), data = pas.plant.beta)</td></tr>
<tr><td>Grassland plant</td><td>Non-native richness</td><td>21.23</td><td>32.46</td><td>0.421</td><td>0.438</td></tr>
<tr><td colspan="6">lmer((value)^(1/3) ~ Pasture_Type + Elevation + (1|Year) + (1|Season), data = pas.plant.inv)</td></tr>
<tr><td>Grassland plant</td><td>ANPP</td><td>95.86</td><td>105.62</td><td>0.168</td><td>0.237</td></tr>
<tr><td colspan="6">lmer(log(value) ~ Pasture_Type + Elevation + (1|Year), data = pas.product)</td></tr>
<tr><td>Grassland plant</td><td>Root biomass</td><td>299.16</td><td>314.01</td><td>0.189</td><td>0.189</td></tr>
<tr><td colspan="6">lmer(sqrt(value) ~ Pasture_Type + Elevation + (1|Year), data = pas.root)</td></tr>
<tr><td>Grassland forage</td><td>Palatable biomass</td><td>679.33</td><td>692.16</td><td>0.433</td><td>0.465</td></tr>
<tr><td colspan="6">lmer(value ~ Pasture_Type + Elevation + (1|Year:Season), data = pas.pala.cover)</td></tr>
<tr><td>Grassland forage</td><td>N</td><td>-398.25</td><td>-376.31</td><td>0.034</td><td>0.411</td></tr>
<tr><td colspan="6">lmer(sqrt(value) ~ Pasture_Type + Elevation + (1|Year/Month), data = pas.forage.ndm)</td></tr>
<tr><td>Grassland forage</td><td>P</td><td>-912.92</td><td>-891.02</td><td>0.069</td><td>0.478</td></tr>
<tr><td colspan="6">lmer(value ~ Pasture_Type + Elevation + (1|Year/Month), data = pas.forage.pdm)</td></tr>
</tbody>
</table>

<table>
<tr><td>Grassland forage</td><td>IVOMD</td><td>1920.9</td><td>1942.8</td><td>0.027</td><td>0.559</td></tr>
<tr><td colspan="6">lmer(value ~ Pasture_Type + Elevation + (1|Year/Month), data = pas.forage.ivomd)</td></tr>
<tr><td>Cattle production</td><td>Stocking density</td><td>694.50</td><td>714.04</td><td>0.289</td><td>0.581</td></tr>
<tr><td colspan="6">lmer(sqrt(value) ~ Pasture_Type + (1+Pasture_Type|Year:Month), data = stocking.density)</td></tr>
<tr><td>Grassland vertebrate</td><td>Native richness</td><td>148.49</td><td>153.85</td><td>0.043</td><td>0.043</td></tr>
<tr><td colspan="6">lm(richness ~ Pasture_Type, data = vert.native)</td></tr>
<tr><td>Grassland vertebrate</td><td>Native diversity</td><td>-48.11</td><td>-42.76</td><td>0.067</td><td>0.067</td></tr>
<tr><td colspan="6">lm(shannon ~ Pasture_Type, data = vert.native)</td></tr>
<tr><td>Grassland vertebrate</td><td>Non-native diversity</td><td>-60.95</td><td>-55.60</td><td>0.065</td><td>0.065</td></tr>
<tr><td colspan="6">lm((1/shannon) ~ Pasture_Type, data = vert.non)</td></tr>
<tr><td>Grassland gas</td><td>CO_2</td><td>695.41</td><td>703.51</td><td>0.003</td><td>0.485</td></tr>
<tr><td colspan="6">lmer(value ~ Pasture_Type + (1|Month), data = EC.gas.co2)</td></tr>
<tr><td>Grassland gas</td><td>CH_4</td><td>131.30</td><td>139.40</td><td>0.093</td><td>0.553</td></tr>
<tr><td colspan="6">lmer(sqrt(value) ~ Pasture_Type + (1|Month), data = EC.gas.ch4)</td></tr>
<tr><td colspan="6">Wetland best models</td></tr>
<tr><td>Wetland soil</td><td>TN</td><td>-70.99</td><td>-60.52</td><td>0.068</td><td>0.124</td></tr>
<tr><td colspan="6">lmer(value ~ Pasture_Type + Elevation + (1|Year), data = wet.soil.tn)</td></tr>
<tr><td>Wetland soil</td><td>TP</td><td>150.18</td><td>160.65</td><td>0.064</td><td>0.600</td></tr>
<tr><td colspan="6">lmer(log(value) ~ Pasture_Type + Elevation + (1|Year), data = wet.soil.tp)</td></tr>
<tr><td>Wetland soil</td><td>C/N ratio</td><td>224.79</td><td>235.26</td><td>0.328</td><td>0.487</td></tr>
<tr><td colspan="6">lmer(value ~ Pasture_Type + Elevation + (1|Year), data = wet.soil.cn)</td></tr>
<tr><td>Wetland soil</td><td>OM</td><td>104.47</td><td>114.94</td><td>0.107</td><td>0.115</td></tr>
<tr><td colspan="6">lmer(log(value) ~ Pasture_Type + Elevation + (1|Year), data = wet.soil.om)</td></tr>
<tr><td>Wetland soil</td><td>TC</td><td>97.70</td><td>108.17</td><td>0.112</td><td>0.112</td></tr>
<tr><td colspan="6">lmer(log(value) ~ Pasture_Type + Elevation + (1|Year), data = wet.soil.tc)</td></tr>
<tr><td>Wetland water</td><td>TN</td><td>-3.36</td><td>8.55</td><td>0.088</td><td>0.338</td></tr>
<tr><td colspan="6">lmer(sqrt(value) ~ Pasture_Type + Elevation + (1|Year), data = wet.water.tn)</td></tr>
<tr><td>Wetland water</td><td>NH_4^+</td><td>-123.42</td><td>-111.57</td><td>0.023</td><td>0.269</td></tr>
<tr><td colspan="6">lmer(sqrt(value) ~ Pasture_Type + Elevation + (1|Year), data = wet.water.nh4)</td></tr>
<tr><td>Wetland water</td><td>NO_3^-</td><td>-103.26</td><td>-93.13</td><td>0.019</td><td>0.513</td></tr>
<tr><td colspan="6">lmer((value)^(1/3) ~ Pasture_Type + Elevation + (1|Year), data = wet.water.no3)</td></tr>
<tr><td>Wetland water</td><td>TP</td><td>220.79</td><td>232.71</td><td>0.397</td><td>0.397</td></tr>
<tr><td colspan="6">lmer(log(value) ~ Pasture_Type + Elevation + (1|Year), data = wet.water.tp)</td></tr>
<tr><td>Wetland water</td><td>PO_4^{3-}</td><td>15.81</td><td>27.72</td><td>0.190</td><td>0.302</td></tr>
<tr><td colspan="6">lmer(sqrt(value) ~ Pasture_Type + Elevation + (1|Year), data = wet.water.op)</td></tr>
<tr><td>Wetland plant</td><td>Total richness</td><td>1600.2</td><td>1617.6</td><td>0.195</td><td>0.254</td></tr>
<tr><td colspan="6">lmer(value ~ Pasture_Type + Elevation + (1|Year), data = wet.plant.tsr)</td></tr>
<tr><td>Wetland plant</td><td>α-diversity</td><td>391.5</td><td>408.9</td><td>0.221</td><td>0.257</td></tr>
<tr><td colspan="6">lmer(sqrt(value) ~ Pasture_Type + Elevation + (1|Year), data = wet.plant.shan)</td></tr>
<tr><td>Wetland plant</td><td>Non-native richness</td><td>361.09</td><td>378.49</td><td>0.481</td><td>0.481</td></tr>
<tr><td colspan="6">lmer(sqrt(value) ~ Pasture_Type + Elevation + (1|Year), data = wet.plant.inv)</td></tr>
<tr><td>Wetland plant</td><td>ANPP</td><td>276.97</td><td>285.29</td><td>0.108</td><td>0.250</td></tr>
<tr><td colspan="6">lmer(sqrt(value) ~ Pasture_Type + Elevation + (1|Year), data = wet.product)</td></tr>
<tr><td>Wetland plant</td><td>Root biomass</td><td>74.54</td><td>80.63</td><td>0.095</td><td>0.843</td></tr>
<tr><td colspan="6">lmer(log(value) ~ Pasture_Type + (1|Soil_Depth)+ (1|Root_Size), data = wet.root)</td></tr>
<tr><td>Wetland forage</td><td>P</td><td>370.78</td><td>394.42</td><td>0.342</td><td>0.768</td></tr>
<tr><td colspan="6">lmer(log(value) ~ Pasture_Type + Elevation + (1|Species) + (1|Year), data = wet.forage.p)</td></tr>
<tr><td>Wetland forage</td><td>N</td><td>217.91</td><td>245.92</td><td>0.012</td><td>0.528</td></tr>
<tr><td colspan="6">lmer(log(value) ~ Pasture_Type + Elevation + (1+Pasture_Type|Species), data = wet.forage.n)</td></tr>
<tr><td>Wetland forage</td><td>C/N ratio</td><td>250.60</td><td>278.61</td><td>0.013</td><td>0.562</td></tr>
<tr><td colspan="6">lmer(log(value) ~ Pasture_Type + Elevation + (1+Pasture_Type|Species), data = wet.forage.cn)</td></tr>
</table>

Wetland forage	Palatable biomass	326.07	334.51	0.404	0.404
lmer(value ~ Pasture_Type + Elevation + (1|Year), data = wet.pala.cover)					
Wetland invertebrate	Richness	8.71	12.69	0.052	0.052
lm(log(value) ~ Pasture_Type + Elevation, data = wet.invert.rich)					
Wetland invertebrate	α-diversity	-40.65	-36.67	0.124	0.124
lm(sqrt(value) ~ Pasture_Type + Elevation, data = wet.invert.shan)					
Wetland animal	Vertebrate richness	87.63	98.10	0.055	0.087
lmer(sqrt(value) ~ Pasture_Type + Elevation + (1|Year), data = wet.vert.rich)					
Wetland animal	Vertebrate diversity	50.60	61.07	0.077	0.120
lmer(value ~ Pasture_Type + Elevation + (1|Year), data = wet.vert.shan)					
Wetland gas	CH_4	148.93	162.82	0.111	0.421
lmer((value)^(1/3) ~ Pasture_Type + (1|Location/Season), data = wet.ch4)					

Supplementary Figures

Figure S1. Map of the Archbold Biological Station's Buck Island Ranch, with eight experimental pastures (indicated as yellow polygons), 20 experimental wetlands (indicated as blue circles), eight ditch water sampling sites (indicated as purple triangles), and two eddy covariance towers in Intensified managed and Semi-natural grasslands.

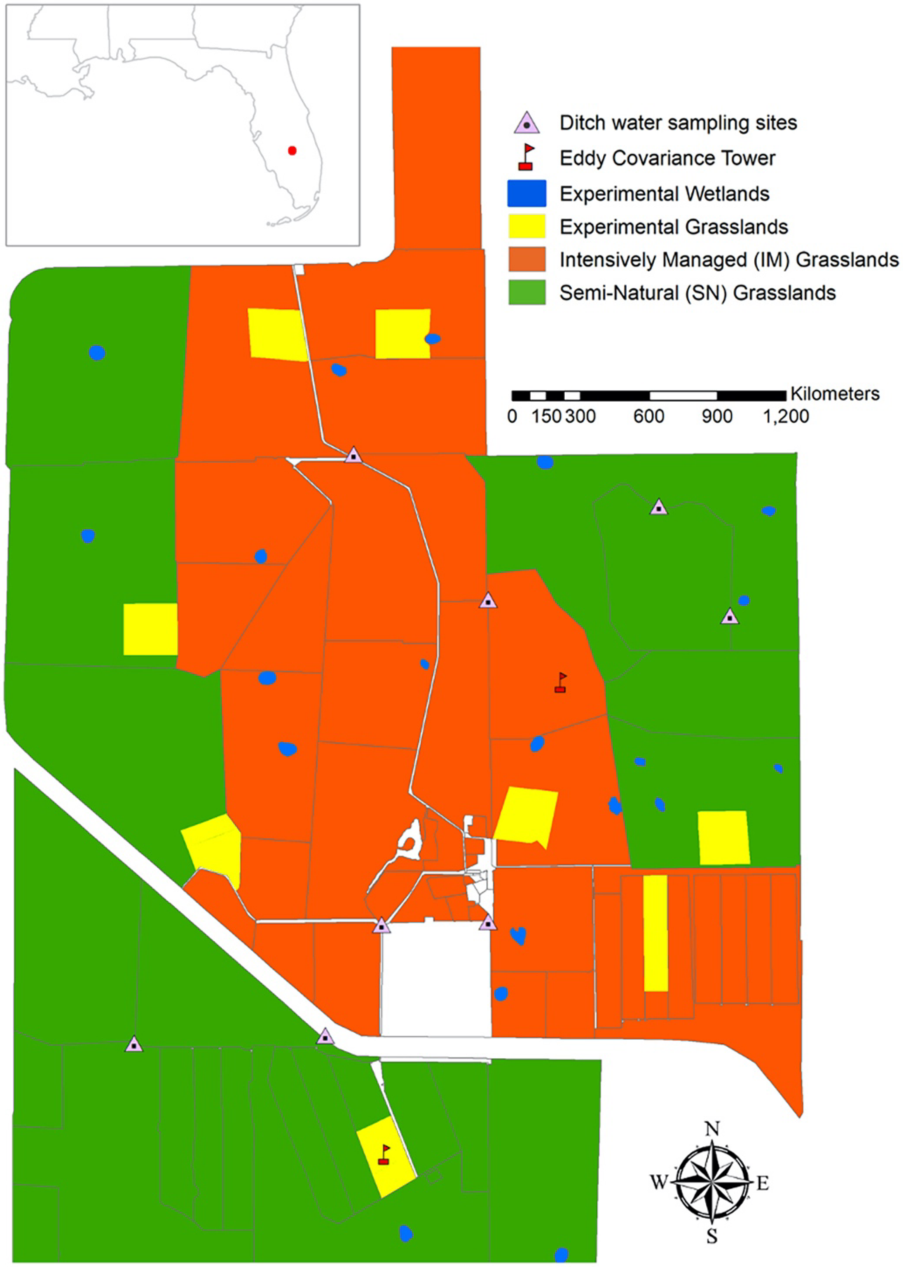

Figure S2. Standardized effect sizes (Hedge's *d*) of land-use intensification effects on total non-native plant richness, planted non-native plant richness, and non-planted non-native plant richness. Effect sizes of intensification were estimated by comparing Intensively managed (IM) vs. Semi-natural (SN) grasslands, with error bars representing 95% confidence intervals. Positive Hedge's *d* denotes a higher indicator value for IM than SN grasslands. Black bars represent significant differences ($\alpha \leq 0.05$) between IM and SN grasslands. Numbers in parentheses mean the sample size for estimating the effect size of each indicator. Source data are provided as a Source Data file.

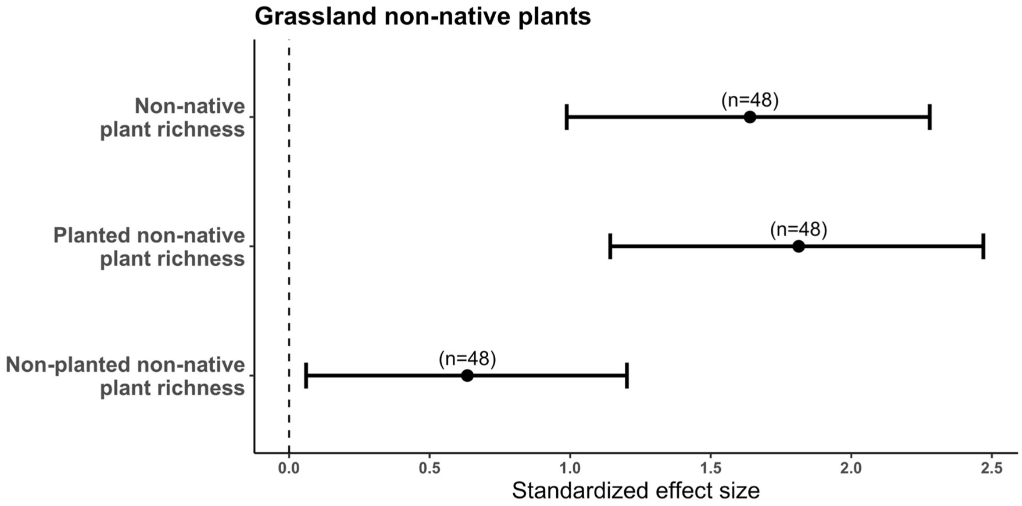

Figure S3. Dendrograms depicting the results of hierarchical cluster analysis, assigning (A) grassland ecosystem service indicators, and (B) wetland ecosystem service indicators to clusters based on their similarities. Source data are provided as a Source Data file.

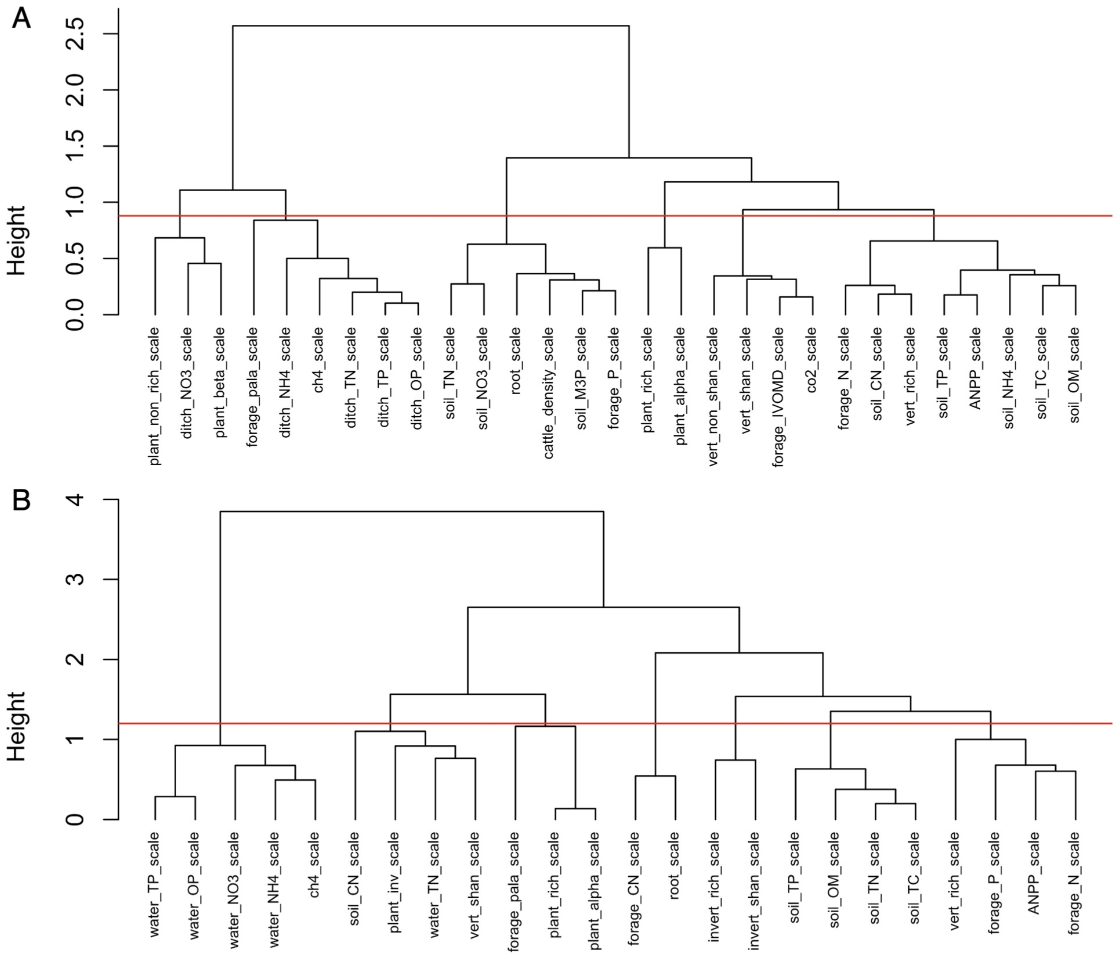

Supplementary References

1. Swain, H. M., Boughton, E. H., Bohlen, P. J. & Lollis, L. O. Trade-offs among ecosystem services and disservices on a Florida ranch. *Rangelands* **35**, 75–87 (2013).
2. Kohmann, M. M. *et al.* Farm-scale phosphorus budgets of beef cow-calf operations. *Nutrient Cycling in Agroecosystems* **119**, 389–403 (2021).
3. Capece, J. C., Campbell, K. L., Bohlen, P. J., Graetz, D. A. & Portier, K. M. Soil phosphorus, cattle stocking rates, and water quality in subtropical pastures in Florida, USA. *Rangeland Ecology & Management* **60**, 19–30 (2007).
4. Boughton, E. H. *et al.* Trade-offs and synergies in a payment-for-ecosystem services program on ranchlands in the Everglades headwaters. *Ecosphere* **10**, e02728 (2019).
5. Gathumbi, S. M., Bohlen, P. J. & Graetz, D. A. Nutrient enrichment of wetland vegetation and sediments in subtropical pastures. *Soil Science Society of America Journal* **69**, 539–548 (2005).
6. Anderson, M. J., Ellingsen, K. E. & McArdle, B. H. Multivariate dispersion as a measure of beta diversity. *Ecology Letters* **9**, 683–693 (2006).
7. Tabak, M. A. *et al.* Machine learning to classify animal species in camera trap images: Applications in ecology. *Methods in Ecology and Evolution* **10**, 585–590 (2019).
8. Knapp, A. K. *et al.* A test of two mechanisms proposed to optimize grassland aboveground primary productivity in response to grazing. *Journal of Plant Ecology* **5**, 357–365 (2012).
9. McNaughton, S. J. Ecology of a grazing ecosystem: The serengeti. *Ecological Monographs* **55**, 259–294 (1985).
10. Boughton, E. H. *et al.* Patch-burn grazing impacts forage resources in subtropical humid grazing lands. *Rangeland Ecology & Management* **84**, 10–21 (2022).
11. Gallaher, R. N., Weldon, C. O. & Futral, J. G. An Aluminum block digester for plant and soil analysis 1. *Soil Science Society of America Journal* **39**, 803–806 (1975).
12. Hambleton, L. G. Semiautomated method for simultaneous determination of phosphorus, calcium, and crude protein in animal feeds. *Journal of the Association of Official Analytical Chemists* **60**, 845–852 (1977).
13. Moore, J. E. & Mott, G. O. Recovery of residual organic matter from in vitro digestion of forages. *Journal of Dairy Science* **57**, 1258–1259 (1974).
14. Paudel, S. *et al.* Intensification differentially affects the delivery of multiple ecosystem services in subtropical and temperate grasslands. *Agriculture, Ecosystems & Environment* **348**, 108398 (2023).
15. Gomez-Casanovas, N. *et al.* Grazing alters net ecosystem C fluxes and the global warming potential of a subtropical pasture. *Ecological Applications* **28**, 557–572 (2018).
16. Ho, J. *et al.* Ranching practices interactively affect soil nutrients in subtropical wetlands. *Agriculture, Ecosystems & Environment* **254**, 130–137 (2018).
17. Jansen, L. S., Pierre, S. & Boughton, E. H. Interactions of fire, grazing and pasture management: Short-term and long-term responses of water quality to management regimes in subtropical isolated wetlands. *Agriculture, Ecosystems & Environment* **280**, 102–113 (2019).

18. Boughton, E. H., Quintana-Ascencio, P. F., Bohlen, P. J., Fauth, J. E. & Jenkins, D. G. Interactive effects of pasture management intensity, release from grazing and prescribed fire on forty subtropical wetland plant assemblages. *Journal of Applied Ecology* **53**, 159–170 (2016).
19. Sonnier, G. *et al.* Pasture management, grazing, and fire interact to determine wetland provisioning in a subtropical agroecosystem. *Ecosphere* **11**, e03209 (2020).
20. Medley, K. A. *et al.* Intense ranchland management tips the balance of regional and local factors affecting wetland community structure. *Agriculture, Ecosystems & Environment* **212**, 207–244 (2015).
21. DeLucia, N. J., Gomez-Casanovas, N., Boughton, E. H. & Bernacchi, C. J. The role of management on methane emissions from subtropical wetlands embedded in agricultural ecosystems. *Journal of Geophysical Research: Biogeosciences* **124**, 2694–2708 (2019).
22. Sonnier, G., Boughton, E. H. & Whittington, R. Long-term response of wetland plant communities to management intensity, grazing abandonment, and prescribed fire. *Ecological Applications* **33**, e2732 (2023).
23. Sonnier, G. *et al.* Pasture management, grazing, and fire interact to determine wetland provisioning in a subtropical agroecosystem. *Ecosphere* **11**, e03209 (2020).
