# Supplementary material for: Grassland intensification effects cascade to alter multifunctionality of wetlands within metaecosystems
Source: Nat Commun. 2023 Dec 13;14:8267. doi: 10.1038/s41467-023-44104-2 (PMC10719369; doi:10.1038/s41467-023-44104-2)
Supplement: Supplementary file 3 — Reporting Summary [file 41467_2023_44104_MOESM3_ESM.pdf]

nature portfolio

Corresponding author(s): Jiangxiao Qiu; Elizabeth Boughton

Last updated by author(s): 11/27/2023

Reporting Summary

Nature Portfolio wishes to improve the reproducibility of the work that we publish. This form provides structure for consistency and transparency in reporting. For further information on Nature Portfolio policies, see our Editorial Policies and the Editorial Policy Checklist.

Statistics

For all statistical analyses, confirm that the following items are present in the figure legend, table legend, main text, or Methods section.

n/a	Confirmed	
☐	☒	The exact sample size (*n*) for each experimental group/condition, given as a discrete number and unit of measurement
☐	☒	A statement on whether measurements were taken from distinct samples or whether the same sample was measured repeatedly
☐	☒	The statistical test(s) used AND whether they are one- or two-sided<br>*Only common tests should be described solely by name; describe more complex techniques in the Methods section.*
☐	☒	A description of all covariates tested
☐	☒	A description of any assumptions or corrections, such as tests of normality and adjustment for multiple comparisons
☐	☒	A full description of the statistical parameters including central tendency (e.g. means) or other basic estimates (e.g. regression coefficient) AND variation (e.g. standard deviation) or associated estimates of uncertainty (e.g. confidence intervals)
☐	☒	For null hypothesis testing, the test statistic (e.g. *F*, *t*, *r*) with confidence intervals, effect sizes, degrees of freedom and *P* value noted<br>*Give P values as exact values whenever suitable.*
☒	☐	For Bayesian analysis, information on the choice of priors and Markov chain Monte Carlo settings
☒	☐	For hierarchical and complex designs, identification of the appropriate level for tests and full reporting of outcomes
☐	☒	Estimates of effect sizes (e.g. Cohen's *d*, Pearson's *r*), indicating how they were calculated

Our web collection on statistics for biologists contains articles on many of the points above.

Software and code

Policy information about availability of computer code

Data collection	All data were collected from field and lab measurements, and no software or code involved
Data analysis	R statistical and R studio are used for data analysis, with all functions and packages publicly available. Code are made freely available.

For manuscripts utilizing custom algorithms or software that are central to the research but not yet described in published literature, software must be made available to editors and reviewers. We strongly encourage code deposition in a community repository (e.g. GitHub). See the Nature Portfolio guidelines for submitting code & software for further information.

Data

Policy information about availability of data

All manuscripts must include a data availability statement. This statement should provide the following information, where applicable:

- Accession codes, unique identifiers, or web links for publicly available datasets
- A description of any restrictions on data availability
- For clinical datasets or third party data, please ensure that the statement adheres to our policy

All data and code are shared as the source data, and all raw data have been deposited into Figshare with free public access and DOI provided in the manuscript.

Human research participants

Policy information about studies involving human research participants and Sex and Gender in Research.

Reporting on sex and gender	No human research involved
Population characteristics	No human research involved
Recruitment	No human research involved
Ethics oversight	No human research involved

Note that full information on the approval of the study protocol must also be provided in the manuscript.

Field-specific reporting

Please select the one below that is the best fit for your research. If you are not sure, read the appropriate sections before making your selection.

☐ Life sciences ☐ Behavioural & social sciences ☒ Ecological, evolutionary & environmental sciences

For a reference copy of the document with all sections, see nature.com/documents/nr-reporting-summary-flat.pdf

Ecological, evolutionary & environmental sciences study design

All studies must disclose on these points even when the disclosure is negative.

Study description	Grassland-wetland mosaic in the subtropical region. The treatment is the effects of agricultural upland intensification, which includes in situ integrated practices including introduction of forage grasses, fertilization, liming, intensive grazing, and extensive ditching in upland grasslands. All detailed study region and experimental designs were elaborated in the online Supplementary Information.
Research sample	We have analyzed and synthesized a long-term of 11,000 field measurements that were collected to capture the multiple dimensions of land intensification effects on ecosystem services in interlinked grasslands and wetlands. The samples aim to represent the system properties and dynamics of subtropical grassland-wetland mosaics.
Sampling strategy	Randomized block sampling, with sample sizes determined based on the representation and coverage of the study systems as well as logistics considerations. All detailed data collection procedures and rationale were elaborated in the online Supplementary Information.
Data collection	Field data collection with hundreds of graduate students, technicians, researchers, and field biologists. All detailed data collection procedures and protocols were elaborated in details in the online Supplementary Information.
Timing and spatial scale	Timing: from 2003-2020 with data collected and aggregated annually to capture long-term dynamics. Spatial scale: point-based measurements across grasslands and wetlands. All details are provided in the online Supplementary Information.
Data exclusions	No data excluded in the analyses.
Reproducibility	Data and Code will be publicly accessible. All code are annotated so as to ensure that results are fully reproducible.
Randomization	Randomization are taking into account at different levels of data collection, such as in selection of sampling sites and sampling location.
Blinding	NA. Because this study is a data synthesis research and not a control experiment. However, for all data used in this data synthesis, randomization was used as a critical criteria to guide our sampling strategy.

Did the study involve field work? ☐ Yes ☒ No

Reporting for specific materials, systems and methods

We require information from authors about some types of materials, experimental systems and methods used in many studies. Here, indicate whether each material, system or method listed is relevant to your study. If you are not sure if a list item applies to your research, read the appropriate section before selecting a response.

Materials & experimental systems

n/a	Involved in the study
☒	☐ Antibodies
☒	☐ Eukaryotic cell lines
☒	☐ Palaeontology and archaeology
☒	☐ Animals and other organisms
☒	☐ Clinical data
☒	☐ Dual use research of concern

Methods

n/a	Involved in the study
☒	☐ ChIP-seq
☒	☐ Flow cytometry
☒	☐ MRI-based neuroimaging
